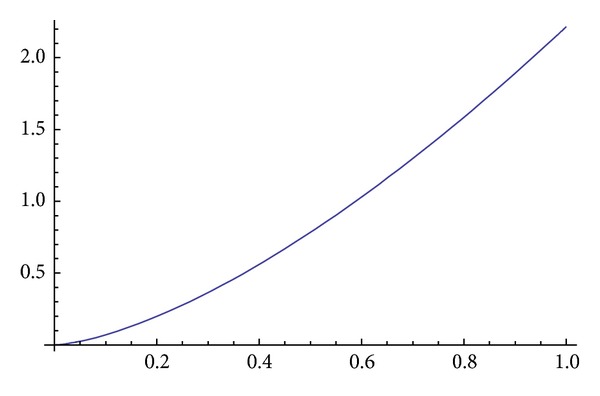# Fractional Solutions of Bessel Equation with *N*-Method

**DOI:** 10.1155/2013/685695

**Published:** 2013-08-19

**Authors:** Erdal Bas, Resat Yilmazer, Etibar Panakhov

**Affiliations:** Department of Mathematics, Firat University, 23119 Elazig, Turkey

## Abstract

This paper deals with the design fractional solution of Bessel equation. We obtain explicit solutions of the equation with the help of fractional calculus techniques. Using the *N*-fractional calculus operator *N* ^*ν*^ method, we derive the fractional solutions of the equation.

## 1. Introduction, **Definitions**, and Preliminaries

 Fractional calculus has an important place in the field of math. Firstly, L'Hospital and Leibniz were interested in the topic in 1695, [1]. Fractional calculus is an area of applied mathematics that deals with derivatives and integrals of arbitrary orders and their applications in science, engineering, mathematics, economics, and other fields. The seeds of fractional derivatives were planted over 300 years ago. Since then many efficient mathematicians of their times, such as N. H. Abel, M. Caputo, L. Euler, J. Fourier, A. K. Grunwald, J. Hadamard, G. H. Hardy, O. Heaviside, H. J. Holmgren, P. S. Laplace, G. W. Leibniz, A. V. Letnikov, J. Liouville, and B. Riemann, have contributed to this field; all these references can be seen in [1–5]. The mathematics involved appeared very different applications of this field. Fractional calculus has been applied to almost every field of science. They are viscoelasticity, electrical engineering, electrochemistry, biology, biophysics and bioengineering, signal and image processing, mechanics, mechatronics, physics, and control theory. During the last decade, Samko et al. [4], Nishimoto [6–12], and Podlubny [3] have been helpful in introducing the field to engineering, science, economics and finance, and pure and applied field. Furthermore, there were many studies in this field [5, 13–16]. Various scientists have studied that concept. The progress in this field continues [2–4, 6–12, 17–20].

*N*-Fractional calculus is a very interesting method because this method is applied to singular equation. Note that fractional solutions can be obtained for kinds of singular equation via this method [6–12, 17]. In this paper, our aim is to apply the same way for singular Sturm-Liouville equation with Bessel potential and find fractional solutions of this equation. Furthermore, we give some applications and their graphs of fractional solutions of the equation.

Now, consider the following the Bessel equation:

(1) $$\begin{array}{c} x\frac{d^{2}z}{dx^{2}}+\frac{dz}{dx}+\left[\lambda x-\frac{p^{2}}{x}\right]z=0,\;0<x\leq 1, \end{array}$$

where *λ* and *p* are real numbers. By means of the substitution $y=\sqrt{xz}$ (1) reduces to the form

(2) $$\begin{array}{c} \frac{d^{2}y}{dx^{2}}+\left(\lambda -\frac{p^{2}-1/4}{x^{2}}\right)y=0. \end{array}$$

Bessel equation for having the analogous singularity is given in [21].

The differintegration operators and their generalizations [6–11, 17, 18] have been used to solve some classes of differential equations and fractional differential equations.

Two of the most commonly encountered tools in the theory and applications of fractional calculus are provided by the Riemann-Liouville operator *R*_*z*_^*υ*^  (*υ* ∈ *ℂ*) and the Weyl operator *W*_*z*_^*υ*^  (*υ* ∈ *ℂ*), which are defined by [17, 19].

Consider

(3) Rzυf(z)={1Γ(υ)∫0z(z−t)υ−1f(t)dt(Re(υ)>0),dndznRzυ+nf(z)(−n<Re(υ)≤0;  n∈ℕ),

(4) Wzυf(z)={1Γ(υ)∫z∞(t−z)υ−1f(t)dt(Re(υ)>0),dndznWzυ+nf(z)(−n<Re(υ)≤0;  n∈ℕ)

provided that the defining integrals in (3) and (4) exist, *ℕ* being the set of positive integers.

**Definition 1 (cf. [6–10, 12, 20])**

Let

(5) $$\begin{array}{c} D=\left\{D^{-},D^{+}\right\},\;C=\left\{C^{-},C^{+}\right\}, \end{array}$$

where *C* ^−^ is a curve along the cut joining two points *z* and −*∞* + *i* *Im*⁡(*z*),  *C* ^+^ is a curve along the cut joining two points *z* and *∞* + *i* *Im*⁡(*z*),  *D* ^−^ is a domain surrounded by *C* ^−^, and *D* ^+^ is a domain surrounded by *C* ^+^. (Here *D* contains the points over the curve *C*.)

Moreover, let *f* = *f*(*z*) be a regular function in *D*  (*z* ∈ *D*),

(6) fν(z)=(f(z))ν=Γ(ν+1)2πi∫Cf(t)dt(t−z)ν+1   (ν∈ℝ∖ℤ−;ℤ−={…,−3,−2,−1}),f−n(z)=lim⁡ν→−nfν(z)        (n∈ℤ+),    

where *t* ≠ *z*,

(7) $$\begin{array}{c} -\pi \leq \arg\left(t-z\right)\leq \pi \;\text{for}\;\;C^{-},\\ 0\leq \arg\left(t-z\right)\leq 2\pi \;\text{for}\;\;C^{+}. \end{array}$$

Then *f*_*ν*_(*z*)  (*ν* > 0) is said to be the fractional derivative of *f*(*z*) of order *ν* and *f*_*ν*_(*z*)  (*ν* < 0) is said to be the fractional integral of *f*(*z*) of order −*ν*, provided (in each case) that |*f*_*ν*_(*z*)| < *∞*  (*ν* ∈ ℝ).

Finally, let the fractional calculus operator (Nishimoto's operator) *N* ^*ν*^ be defined by (cf. [6–10])

(8) $$\begin{array}{c} N^{\nu }=\left(\frac{\Gamma \left(\nu +1\right)}{2\pi i}\int_{C}\frac{dt}{{\left(t-z\right)}^{\nu +1}}\right)\;\left(\nu \notin {\mathbb{Z}}^{-}\right) \end{array}$$

with

(9) $$\begin{array}{c} N^{-n}=\underset{\nu \to -n}{\lim}N^{\nu }\;\left(n\in {\mathbb{Z}}^{+}\right). \end{array}$$

We find it to be worthwhile to recall here the following useful lemmas and properties associated with the fractional differintegration which was defined earlier (cf. e.g., [6–10, 12]).

**Lemma 2 (linearity property)**

If the functions *f*(*z*) and *g*(*z*) are single-valued and analytic in some domain *Ω*⊆*ℂ*, then

(10) (h1f(z)+h2g(z))ν=h1fν(z)+h2gν(z)                (ν∈ℝ;z∈Ω)

for any constants *h*_1_ and *h*_2_.

**Lemma 3 (index law)**

If the function *f*(*z*) is single-valued and analytic in some domain *Ω*⊆*ℂ*, then

(11) (fσ(z))ν=fσ+ν(z)=(fν(z))σ(fσ(z)≠0;fν(z)≠0;σ,ν∈ℝ;z∈Ω).

**Lemma 4 (generalized Leibniz rule)**

If the functions *f*(*z*) and *g*(*z*) are single-valued and analytic in some domain *Ω*⊆*ℂ*, then

(12) (f(z)·g(z))ν=∑n=0∞(νn)fν−n(z)·gn(z)             (ν∈ℝ;z∈Ω),

where *g*_*n*_(*z*) is the ordinary derivative of *g*(*z*) of order *n*  (*n* ∈ *ℕ*_0_ : = *ℕ* ∪ {0}), being tacitly assumed (for simplicity) that *g*(*z*) is the polynomial part (if any) of the product *f*(*z*)*g*(*z*).

**Property 1**

For a constant *λ*,

(13) $$\begin{array}{c} {\left(e^{\lambda z}\right)}_{\nu }=\lambda^{\nu }e^{\lambda z}\;\left(\lambda \neq 0;\nu \in \mathbb{R};z\in \mathbb{C}\right). \end{array}$$

**Property 2**

For a constant *λ*,

(14) $$\begin{array}{c} {\left(e^{-\lambda z}\right)}_{\nu }=e^{-i\pi \nu }\lambda^{\nu }e^{-\lambda z}\;\left(\lambda \neq 0;\nu \in \mathbb{R};z\in \mathbb{C}\right). \end{array}$$

**Property 3**

For a constant *λ*,

(15) (zλ)ν=e−iπνΓ(ν−λ)Γ(−λ)zλ−ν(ν∈ℝ;z∈ℂ;|Γ(ν−λ)Γ(−λ)|<∞).

Now, let apply *N*-fractional method to nonhomogeneous Bessel equation.

## 2. The *N* ^*ν*^-Method Applied to Bessel Equation

**Theorem 5**

Let *y* ∈ {*y* : 0 ≠ |*y*_*ν*_| < *∞*; *ν* ∈ ℝ} and *f* ∈ {*f* : 0 ≠ |*f*_*ν*_| < *∞*; *ν* ∈ ℝ}. We consider the nonhomogeneous Bessel equation:

(16) $$\begin{array}{c} L\left[y,x,\lambda ,p\right]=y_{2}+y\left[\lambda -\frac{p^{2}-1/4}{x^{2}}\right]=f\;\left(0<x\leq 1\right), \end{array}$$

and it has particular solutions of the forms

(17) yı=xp+1/2e−iλx×{[(fx1/2−peiλx)−p−1/2e−2iλxxp−1/2]−1  ×e2iλxx−p−1/2}p−1/2,

(18) yıı=xp+1/2eiλx×{[(fx1/2−pe−iλx)−p−1/2e2iλxxp−1/2]−1  ×e−2iλxx−p−1/2}p−1/2,

(19) yııı=x−p+1/2e−iλx×{[(fx1/2+peiλx)p−1/2e−2iλxx−p−1/2]−1  ×e2iλxxp−1/2}−p−1/2,

(20) yıv=x−p+1/2eiλx×{[(fx1/2+pe−iλx)p−1/2e2iλxx−p−1/2]−1  ×e−2iλxxp−1/2}−p−1/2,

where *y*_2_ = *d* ^2^ *y*/*dx* ^2^,  *y* = *y* (*z*)  (*z* ∈ *ℂ*),  *f* = *f*(*z*) (an arbitrary given function), and *p*, *λ* are given constants.

**Remark 6**

The cases *p* = 0 of (19) and (20) coincide with those (17) and (18).

**Proof**

Set

(21) $$\begin{array}{c} y=x^{\eta }\psi ,\;\psi =\psi \left(x\right). \end{array}$$

Thus

(22) $$\begin{array}{c} y_{1}=\eta x^{\eta -1}\psi +x^{\eta }\psi_{1},\\ y_{2}=\eta \left(\eta -1\right)x^{\eta -2}\psi +2\eta x^{\eta -1}\psi_{1}+x^{\eta }\psi_{2}. \end{array}$$

Putting (21) and (22) in (16), we obtained

(23) ψ2xη+ψ12ηxη−1+ψη(η−1)xη−2+xηψ[λ−p2−1/4x2]=f.

With some rearrangement of the terms in (23), we have

(24) $$\begin{array}{c} \psi_{2}x+\psi_{1}2\eta +\psi \left[x^{-1}\left(\eta^{2}-\eta +\frac{1}{4}-p^{2}\right)+\lambda x\right]=fx^{1-\eta }. \end{array}$$

Here, we choose *η* such that

(25) $$\begin{array}{c} \eta^{2}-\eta +\frac{1}{4}-p^{2}=0. \end{array}$$

That is,

(26) $$\begin{array}{c} \eta =\frac{1}{2}\pm p. \end{array}$$

(I) Let *η* = *p* + (1/2). From (21) and (24), we have

(27) $$\begin{array}{c} y=x^{p+(1/2)}\psi , \end{array}$$

(28) $$\begin{array}{c} \psi_{2}x+\psi_{1}\left(2p+1\right)+\psi \lambda x=fx^{(1/2)-p}. \end{array}$$

Set

(29) $$\begin{array}{c} \psi =e^{\mu x}\phi ,\;\;\;\phi =\phi \left(x\right).\;\;\;\; \end{array}$$

Rewrite (28) in the form

(30) $$\begin{array}{c} {\left(e^{\mu x}\phi \right)}_{2}x+{\left(e^{\mu x}\phi \right)}_{1}\left(2p+1\right)+e^{\mu x}\phi \lambda x=fx^{(1/2)-p}. \end{array}$$

At this point, differentiating *e* ^*μx*^ *ϕ* two times,

(31) $$\begin{array}{c} {\left(e^{\mu x}\phi \right)}_{1}=e^{\mu x}\left(\mu \phi +\phi_{1}\right),\\ {\left(e^{\mu x}\phi \right)}_{2}=e^{\mu x}\left(\mu^{2}\phi +2\mu \phi_{1}+\phi_{2}\right), \end{array}$$

and substituting from (29) and (31) in (30), we can express (30) as

(32) ϕ2x+ϕ1(2μx+2p+1) +ϕ(2pμ+μ+x(μ2+λ))=fx(1/2)−pe−μx.

Choose *μ* such that

(33) $$\begin{array}{c} \mu^{2}+\lambda =0. \end{array}$$

That is,

(34) $$\begin{array}{c} \mu =\pm \sqrt{\lambda }i. \end{array}$$

(I) (i): For instance, taking $\mu =-\sqrt{\lambda }i$, we have

(35) $$\begin{array}{c} \psi =e^{-\sqrt{\lambda }ix}\phi , \end{array}$$

(36) ϕ2x+ϕ1[−λi2x+2p+1] +ϕ[−i(2p+1)λ]=fx(1/2)−pe−μx

from (29) and (32).

Applying the operator *N* ^*ν*^ to both members of (36), we find the following equality:

(37) [ϕ2x]ν+{ϕ1[−λi2x+2p+1]}ν +{ϕ[−i(2p+1)λ]}ν=[fx(1/2)−pe−μx]ν.

Using (3)–(12), we have

(38) [ϕ2x]ν=xϕ2+ν+νϕ1+ν,{ϕ1[−λi2x+2p+1]}ν =ϕ1+ν[−λi2x+2p+1]−λi2νϕν.

Making use of the relations (38), rewrite (37) in the following form:

(39) ϕ2+νx+ϕ1+ν[−λi2x+2p+1+ν] −ϕν[λi2ν+i(2p+1)λ]=[fx(1/2)−peiλx]ν.

Choose *ν* such that

(40) $$\begin{array}{c} \nu =-p-\frac{1}{2}. \end{array}$$

We then have

(41) ϕ−p+3/2x+ϕ−p+1/2[p+12−2λix] =[fx(1/2)−peiλx]−p−1/2

from (39).

Next, writing

(42) $$\begin{array}{c} \phi_{-p+1/2}=\omega =\omega \left(x\right), \end{array}$$

we obtain the following equality from (41):

(43) ω1+ω[x−1(p+12)−2λi] =[fx(1/2)−peiλx]−p−1/2x−1.

This is an ordinary differential equation of the first order which has a particular solution,

(44) ω=[[fx(1/2)−peiλx]−p−1/2x−1e−2λixxp+1/2]−1 ×e2λixx−p−1/2.

Making use of the reverse process to obtain *y* ^*ı*^, we finally obtain the solution (17) from (44), (42), (35), and (27). 

Inversely, (44) satisfies (43); then

(45) $$\begin{array}{c} \phi =\omega_{p-1/2} \end{array}$$

satisfies (41). Therefore, (17) satisfies (16) because we have (27), (35), (44), and (45).

(I) (ii): In the case when $\mu =\sqrt{\lambda }i$, we have

(46) $$\begin{array}{c} \psi =e^{\sqrt{\lambda }ix}\phi , \end{array}$$

(47) ϕ2x+ϕ1[2λix+2p+1] +ϕ[(2p+1)λi]=fx(1/2)−pe−iλx

from (29) and (32).

Applying the operator *N* ^*ν*^ to both members of (47), we have

(48) ϕ2+νx+ϕ1+ν[2λix+2p+1+ν] +ϕν[λi(2p+1)i]=(fx(1/2)−pe−iλx)ν.

Choosing *ν* such that

(49) $$\begin{array}{c} \nu =-p-\frac{1}{2} \end{array}$$

and replacing

(50) $$\begin{array}{c} \phi_{-p+1/2}=\vartheta =\vartheta \left(x\right), \end{array}$$

we then obtain

(51) ϑ1+ϑ[2λi+(p+12)x−1] =(fx(1/2)−pe−iλx)−p−1/2x−1

from (48). A particular solution of (51) is given by

(52) ϑ=[(fx1/2−pe−iλx)−p−1/2x−1e2λixxp+1/2dx]−1 ×e−2λixx−p−1/2.

Thus, we have (18) from (52), (50), (46), and (27).

(II) Let *η* = −*p* + (1/2).

With the help of the similar method in (I), replacing *p* by −*p* in (I) (i) and (I) (ii), we have other solutions (19) and (20) different from (17) and (18), respectively, if *p* ≠ 0.

## 3. The Operator *N* ^*ν*^-Method to a Homogeneous Bessel Equation

**Theorem 7**

If $y\in \overset{o}{℘,}$ just as in Theorem 5, then the homogeneous Bessel equation

(53) L[y,x,p]=y2+y[λ−p2−1/4x2]=0,           (0<x≤1),

has solutions of the forms

(54) $$\begin{array}{c} y_{ı}=kx^{p+1/2}e^{-i\sqrt{\lambda }x}{\left\{e^{2i\sqrt{\lambda }x}x^{-p-1/2}\right\}}_{p-1/2}, \end{array}$$

(55) $$\begin{array}{c} y_{ıı}=kx^{p+1/2}e^{i\sqrt{\lambda }x}{\left\{e^{-2i\sqrt{\lambda }x}x^{-p-1/2}\right\}}_{p-1/2}, \end{array}$$

(56) $$\begin{array}{c} y_{ııı}=kx^{-p+1/2}e^{-i\sqrt{\lambda }x}{\left\{e^{2i\sqrt{\lambda }x}x^{p-1/2}\right\}}_{-p-1/2}, \end{array}$$

(57) $$\begin{array}{c} y_{ıv}=kx^{-p+1/2}e^{i\sqrt{\lambda }x}{\left\{e^{-2i\sqrt{\lambda }x}x^{p-1/2}\right\}}_{-p-1/2}, \end{array}$$

for *p* ≠ 0, where *k* is an arbitrary constant.

**Remark 8**

In the case when *p* = 0, (56) and (57) coincide with (54) and (55).

**Proof**

When *f* = 0 in Section 2, we have

(58) $$\begin{array}{c} \omega_{1}+\omega \left[\left(p+\frac{1}{2}\right)x^{-1}-2\sqrt{\lambda }i\right]=0, \end{array}$$

(59) $$\begin{array}{c} \vartheta_{1}+\vartheta \left[\left(p+\frac{1}{2}\right)x^{-1}+2\sqrt{\lambda }i\right]=0, \end{array}$$

for $\mu =-i\sqrt{\lambda }$ and $\mu =i\sqrt{\lambda }$, instead of (43) and (51).

Therefore, we get (54) for (58) and (55) for (59).

And, for *η* = −*p* + (1/2), replacing *p* by −*p* in (58) and (59), we have (56) and (57).

**Theorem 9**

Let $y\in \overset{o}{℘}$ and $f\in \overset{o}{℘}$ just as in Theorem 5. Then the nonhomogeneous modified Sturm-Liouville equation (16) is satisfied by the fractional differintegrated functions

(60) $$\begin{array}{c} y=y^{ı}+y_{ı}. \end{array}$$

**Proof**

It is clear by Theorems 5 and 7.

*Application 1*. If we substitute *p* = 0,  *λ* = 1/4, and *f* = *ix* ^−1/2^ *e* ^−(*i*/2)*x*^ in (16), then we obtain the following equation:

(61) $$\begin{array}{c} y_{2}+y\left(\frac{1}{4}+\frac{1}{4x^{2}}\right)=ix^{-1/2}e^{-(i/2)x}, \end{array}$$

and its solution is

(62) y=x1/2e(−i/2)x{[(ix−1/2e−(i/2)xx1/2e(i/2)x)−1/2      ×e−ixx−1/2]−1eixx−1/2}−1/2.

By performing the necessary operations in (62), we get

(63) $$\begin{array}{c} y=x^{1/2}e^{(-i/2)x}{\left\{{\left[\frac{2i\sqrt{x}}{\sqrt{\pi }}e^{-ix}x^{-1/2}\right]}_{-1}e^{ix}x^{-1/2}\right\}}_{-1/2}, \end{array}$$

where Riemann Liouville operator is

(64) $$\begin{array}{c} {\left[i\right]}_{-1/2}=\frac{1}{\Gamma \left(1/2\right)}\int_{0}^{x}\frac{i}{\sqrt{x-t}}dt=\frac{2i\sqrt{x}}{\sqrt{\pi }},\\ y=x^{1/2}e^{(-i/2)x}{\left(-\frac{2x^{-1/2}}{\sqrt{\pi }}\right)}_{-1/2}, \end{array}$$

and using the definitions of Riemann Liouville operator again, we obtain the following solution:

(65) $$\begin{array}{c} {\left(-\frac{2x^{-1/2}}{\sqrt{\pi }}\right)}_{-1/2}=-\frac{1}{\Gamma \left(1/2\right)}\int_{0}^{x}\frac{2t^{-1/2}}{\sqrt{\pi }\sqrt{x-t}}dt=-2, \end{array}$$

(66) $$\begin{array}{c} y=-2x^{1/2}e^{-(i/2)x}. \end{array}$$

Now, let us show that the last equality is the solution of (61):

(67) $$\begin{array}{c} y_{2}=e^{-(i/2)x}\left[\frac{1}{2}x^{1/2}+\frac{1}{2}x^{-3/2}+ix^{-1/2}\right]. \end{array}$$

**Figure 1:**
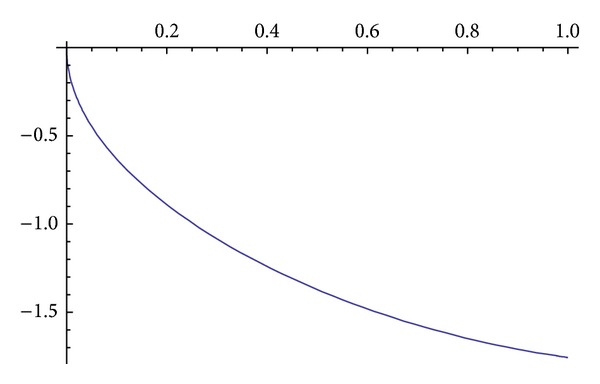
Rey.

**Figure 2:**
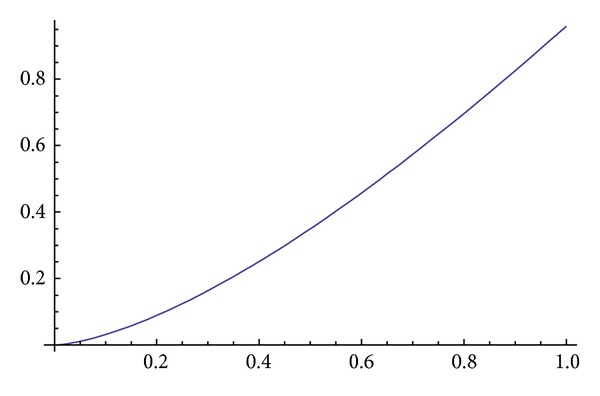
Imy.

Obviously, if (66) and (67) are put in (61), it is satisfied. The graph of the solution of (61) is given in Figures 1 and 2.

*Application* *2*. If we substitute *p* = −1 and  *λ* = 0 in (53), then we obtain the following equation:

(68) $$\begin{array}{c} y_{2}-\frac{3}{4x^{2}}y=0\;\left(0<x\leq 1\right), \end{array}$$

and its solution is

(69) $$\begin{array}{c} y=kx^{-1/2}{\left\{x^{1/2}\right\}}_{-3/2}.\;\;\;\;\;\;\;\; \end{array}$$

We prove that *y*_*ı*_ is the solution of (67). With the help of Riemann Liouville operator,

(70) $$\begin{array}{c} {\left[x^{-1/2}\right]}_{-1/2}=\frac{1}{\Gamma \left(3/2\right)}\int_{0}^{x}\frac{t^{1/2}}{\sqrt{x-t}}dt=\frac{\sqrt{\pi }x^{2}}{4}, \end{array}$$

(71) $$\begin{array}{c} y=\frac{kx^{3/2}\sqrt{\pi }}{4}. \end{array}$$

Now, let us show that the last equality is the solution of (67),

(72) $$\begin{array}{c} y_{2}=\frac{3kx^{-1/2}\sqrt{\pi }}{16}. \end{array}$$

Obviously, if (71) and (72) are put into (68), it is satisfied. The graph of the solution of (68) is given in Figure 3.

## 4. Two Further Cases of Modified Bessel Equation

**Theorem 10**

In the similar way as in the previous sections, we can solve the following nonhomogeneous modified Bessel equation:

(73) $$\begin{array}{c} y_{2}+y\left[\lambda +\frac{\left(1/4\right)+p^{2}}{x^{2}}\right]=f,\\ y_{2}+y\left[-\lambda +\frac{\left(1/4\right)+p^{2}}{x^{2}}\right]=f, \end{array}$$

which are obtained by replacing *p* by *ip* (−*λ* instead of *λ*) in (16); that is,

(74) $$\begin{array}{c} y_{2}+y\left[\lambda +\frac{\left(1/4\right)-{\left(ip\right)}^{2}}{x^{2}}\right]=f, \end{array}$$

(75) $$\begin{array}{c} y_{2}+y\left[-\lambda +\frac{\left(1/4\right)-{\left(ip\right)}^{2}}{x^{2}}\right]=f. \end{array}$$

(i) Therefore, the solutions for (74) are given by replacing *p* by *ip* in (17), (18), (19), and (20) as follows:

(76) y(ı)=xpi+1/2e−iλx×{[(fx1/2−pieiλx)−pi−1/2e−2iλxxpi−1/2]−1  ×e2iλxx−pi−1/2}pi−1/2,y(ıı)=xpi+1/2eiλx×{[(fx1/2−pie−iλx)−pi−1/2e2iλxxpi−1/2]−1  ×e−2iλxx−pi−1/2}pi−1/2,y(ııı)=x−pi+1/2e−iλx×{[(fx1/2+pieiλx)pi−1/2e−2iλxx−pi−1/2]−1  ×e2iλxxpi−1/2}−pi−1/2,y(ıv)=x−pi+1/2eiλx×{[(fx1/2+pie−iλx)pi−1/2e2iλxx−pi−1/2]−1  ×e−2iλxxpi−1/2}−pi−1/2.

(ii) In the same way, for the solutions for (75), substituting the relations (21), and (22) into (75), we have

(77) $$\begin{array}{c} \phi_{2}x+\phi_{1}2\nu +\phi \left[\left(\nu^{2}-\nu +\frac{1}{4}-{\left(pi\right)}^{2}\right)x^{-1}-\lambda x\right]=fx^{1-\nu }. \end{array}$$

Choose *ν* as follows:

(78) $$\begin{array}{c} \nu^{2}-\nu +\frac{1}{4}+p^{2}=0. \end{array}$$

That is

(79) $$\begin{array}{c} \nu =\frac{1}{2}\pm pi. \end{array}$$

Let *ν* = *pi* + (1/2). From (21) and (77), we have

(80) $$\begin{array}{c} y=x^{pi+(1/2)}\phi , \end{array}$$

(81) $$\begin{array}{c} \phi_{2}x+\phi_{1}\left(2pi+1\right)-\phi \lambda x=fx^{(1/2)-pi}. \end{array}$$

Next, set (29); then (81) is rewritten in the form

(82) $$\begin{array}{c} {\left(e^{\mu x}\psi \right)}_{2}x+{\left(e^{\mu x}\psi \right)}_{1}\left(2ip+1\right)-e^{\mu x}\psi \lambda x=fx^{(1/2)-pi}. \end{array}$$

Substituting the relations (29) and (31) into (82), we have

(83) ψ2x+ψ1(2μx+2pi+1) +ψ[(μ2−λ)x+(2pi+1)μ]=fx(1/2)−ipe−μx.

Choose *μ* as follows:

(84) $$\begin{array}{c} \mu^{2}-\lambda =0. \end{array}$$

That is,

(85) $$\begin{array}{c} \mu =\pm \sqrt{\lambda }. \end{array}$$

(ii. 1) In the case when $\mu =-\sqrt{\lambda }$, we have

(86) $$\begin{array}{c} \phi =e^{-\sqrt{\lambda }x}\psi , \end{array}$$

(87) ψ2x+ψ1(−2λx+2pi+1) −ψ[λ(2pi+1)]=fx(1/2)−pieλx

from (29) and (83).

Applying the operator *N* ^*ν*^ to both members of (87), we then obtain

(88) (ψ2x)ν+[ψ1(−2λx+2pi+1)]ν +{ψ[−λ(2pi+1)]}ν=(fx(1/2)−pieλx)ν.

Using (4), (10), and (11), we have

(89) ψ2+νx+ψ1+ν(−2λx+2pi+1+ν) +ψν[−λ(2pi+1+2v)]=(fx(1/2)−pieλx)ν.

Choose *ν* such that

(90) $$\begin{array}{c} \nu =-pi-\frac{1}{2}. \end{array}$$

We then have

(91) ψ2−pi−1/2x+ψ1−pi−1/2[−2λx+pi+12] =(fx(1/2)−pieλx)−pi−1/2

from (89).

Next, writing

(92) $$\begin{array}{c} \psi_{1/2-pi}=u=u\left(x\right), \end{array}$$

we obtain

(93) u1+u[−2λ+(pi+12)x−1]=(fx(1/2)−pieλx)−pi−1/2x−1

from (91). This is an ordinary differential equation of the first order which has a particular solution

(94) u=[(fx(1/2)−pieλx)−pi−1/2e−2λxxpi−1/2]−1×e2λxx−pi−1/2.

We finally obtain the solution

(95) y(ı)=xpi+1/2e−λx×{[(fx1/2−pieλx)−pi−1/2e−2λxxpi−1/2]−1  ×e2λxx−pi−1/2}pi−1/2.

from (94), (92), (86) and (80).

(ii. 2) Similarly, in the case when $\mu =\sqrt{\lambda }$, we obtain

(96) y(ıı)=rpi+1/2eλx×{[(fx1/2−pie−λx)−pi−1/2e2λxxpi−1/2]−1  ×e−2λxx−pi−1/2}pi−1/2.

Let *ν* = −*ip* + (1/2). In the same way as in the procedure in (ii), replacing *ip* by –*ip*  (ii. 1) and (ii. 2), we can obtain *y* ^(*ııı*)^ and *y* ^(*ıv*)^.

**Theorem 11**

In the homogeneous case for (74) with *f* = 0, using the solutions (54), (55), (56), and (57) and replacing *p* by *pi*, we obtain

(97) $$\begin{array}{c} y_{\left(ı\right)}=\alpha x^{pi+1/2}e^{-i\sqrt{\lambda }x}{\left(e^{2i\sqrt{\lambda }x}x^{-pi-1/2}\right)}_{pi-1/2},\\ y_{\left(ıı\right)}=\alpha x^{pi+1/2}e^{i\sqrt{\lambda }x}{\left(e^{-2i\sqrt{\lambda }x}x^{-pi-1/2}\right)}_{pi-1/2},\\ y_{\left(ııı\right)}=\alpha x^{-pi+1/2}e^{-i\sqrt{\lambda }x}{\left(e^{2i\sqrt{\lambda }x}x^{pi-1/2}\right)}_{-pi-1/2},\\ y_{\left(ıv\right)}=\alpha x^{-pi+1/2}e^{i\sqrt{\lambda }x}{\left(e^{-2i\sqrt{\lambda }x}x^{pi-1/2}\right)}_{-pi-1/2}, \end{array}$$

for *p* ≠ 0, where *α* is an arbitrary constant.

## 5. Conclusion

The *N*-fractional calculus operator *N* ^*ν*^-method is applied to the nonhomogeneous and homogeneous Bessel equation. Explicit fractional solutions of Bessel equations are obtained. Furthermore, similar solutions were obtained for the modified same equation by using the method.

## Figures and Tables

**Figure 3:**